# Proceedings of the International Workshop on Cancer Advocacy for African Countries

**DOI:** 10.1186/1750-9378-8-S1-S1

**Published:** 2013-07-15

**Authors:** Richard Segal, Folakemi T Odedina, Shannon Pressey

**Affiliations:** 1 Department of Pharmaceutical Outcomes and Policy, University of Florida, Gainesville, FL 32610, USA

## Introduction

Non-communicable diseases (NCDs) are estimated to be the leading causes of morbidity and mortality in developing countries, especially Africa [1-3]. About 20 percent of the deaths from NCDs in Africans over the age of 45 years of age is from cancer [4]. Known as Africa’s silent killer, cancer is now a major public health problem in Africa, with the five most frequent cancers being Breast, Cervix, Liver, Prostate and Non-Hodgkin Lymphoma [1]. However, cancer continues to be underestimated and ignored in Africa. The little attention being given to cancer has led to unnecessary deaths and suffering from cancer, indicating a need for cancer advocacy as one of several strategies for creating awareness of cancer in local and national communities and the need to commit resources aimed at achieving cancer control objectives. Unfortunately, cancer advocacy is currently limited and weak in Africa, thereby making cancer issues of low priority in African countries.

A long-term goal of the organizers of the conference is to make cancer a top priority in Africa. In pursuit of this goal, the objective of this workshop was to train cancer advocates who will be empowered to engage their communities, develop, and implement cancer health and survivorship programs. The workshop was designed to provide participants with the skills to: (1) mobilize the resources within African countries for health promotion, prevention, and survivorship strategies; (2) partner with key stakeholders to accomplish targeted objectives; (3) raise funds to support advocacy activities; and (4) develop and successfully organize community-centered programs. The specific objectives for the participants included to:

1. Provide opportunities for mutual learning, knowledge transfer, and collaborations among cancer advocates. Participants were provided with learning opportunities to:

a. Assess and track the cancer health needs of their communities;

b. Conduct a SWOT analysis to develop effective strategies, including the development of a local community action plan;

c. Initiate and develop partnerships with other stakeholders to address the needs of their communities;

d. Organize and implement programs to: (i) raise public awareness about cancer, (ii) provide support to those living with cancer, (iii) help advance cancer research and training, (iv) improve the quality of cancer care, and (v) address legislative and regulatory issues that affect cancer care and research in Africa; and

e. Utilize evaluation tools to determine, document, and improve the program outcomes of programs implemented in their communities.

2. Facilitate networking among individuals involved in cancer control, education and research in Africa.

3. Facilitate the development of a global community of practice to address common challenges in cancer advocacy in Africa.

4. Contribute to a global impact against cancer through advocacy.

The International Workshop on Cancer Advocacy for African Countries (CAAC) was organized by the University of Florida, the Prostate Net^®^ and the African Organisation for Research and Training in Cancer (AORTIC) in partnership with several other organizations including the National Cancer Institute and World Wide Prostate Cancer Coalition. The conference was held in Cairo, Egypt on November 29, 2011. A total of 73 delegates participated in the conference including delegates from Africa, the Caribbean, the United Kingdom, Italy, Germany, Canada, and America. Thirty delegates representing thirteen African countries were selected as recipients of scholarship funding.

The ***Opening Session*** began with opening remarks and a welcoming of the workshop delegates by Professor Folakemi Odedina (Conference Chair) and a program overview provided by Mr. Virgil Simons (Conference Co-Chair). The opening presentation titled “**Setting the Stage for Cancer Advocacy in Africa: What and Why?”** was given by Professor Odedina (USA) and Mr. Simons (USA). Professor Odedina offered insights based on her experiences in Nigeria and elsewhere in Africa in developing cancer advocacy initiatives, including the establishment of a National Cancer Awareness month in Nigeria and other advocacy efforts such as organizing Cancer Control meetings at the local, regional, national and international levels and conducting media programs to educate the public on cancer and raise awareness. Their presentation also covered the principles of advocacy and types of advocacy appropriate for cancer control.

This presentation set the stage for small group R**oundtable Discussions on best practices for cancer advocacy**. This session, presided by Professor Ogunbiyi (Nigeria), involved six roundtables: support advocacy presided by Kandusi (Tanzania), community outreach advocacy presided by Bekele (Ethiopia), education advocacy presided by Kamara (Sierra Leone), political advocacy presided by Daramola (Nigeria), research advocacy presided by Mapara (Zimbabwe), and fundraising advocacy presided by Stevn (South Africa). Following the roundtable discussions, the presiders for each roundtable reported a set of action items resulting from the discussions, which is summarized as part of the proceedings.

Professor Odedina (USA) described the importance of conducting locally-based needs assessments in the area of cancer control and the process used for performing these assessments in her presentation titled “**Understanding Your Community and Assessing your Community Health Assets and Needs.**” Her presentation addressed the actions steps necessary for conducting a needs assessment including assembling the community team, developing a team strategy, reviewing community sectors, gathering, entering, reviewing data, and building a community action plan. She concluded her presentation by reminding the delegates that if the problems are in the community, the solutions are also in the community.

The workshop also examined how cancer advocates may engage politicians and researchers. The keynote presentation titled “**Partnering with Elected Officials to Advocate Cancer-Related Policies**” was given by former Senator Anthony Hill Sr. (Minority Whip, Florida, USA). Senator Hill’s presentation offered insights to the political process in the USA and how cancer advocates can best position their messages for action by politicians. Scott Williams (USA), representing the Men’s Health Network, presided over a **Prostate Cancer Roundtable on the topic of Gaining Political Advantage**. Mr. Williams discussed the role of non-profit organizations in forming health prevention messages and developing initiatives especially in the area of cancer control. This presentation was followed by Professor Tim Rebbeck (USA) and Virgil Simons (USA) discussing the importance for cancer advocates and the research community to develop strategic partnerships in a presentation titled “**Finding the Lethal Phenotype: Research Partnership Needs.”** Professor Rebbeck discussed what is needed to develop research capacity in Africa, emphasizing the need for dedicated influential local principal investigators (PIs), an environment that fosters PIs, research goals which are achievable with a realistic timeframe, and strong mentors with bi-directional visits between mentors and local PIs.

In the next session, Professor Renee Reams (USA) described methods she has used to engage the media to promote health prevention initiatives in a presentation titled “**Engaging the Media & Promoting Your Program**.” The next discussion was led by Professor Folakemi Odedina and Mr. Virgil Simons on the formation of the African Cancer Advocacy Consortium (ACAC) at this workshop. They noted that the mission is making cancer a top priority in Africa and described the organization of ACAC, noting six types of advocacy groups comprising ACAC: (1) political advocacy, (2) education advocacy, (3) research advocacy, (4) fundraising advocacy, (5) support advocacy, and (6) community outreach advocacy. An interim ACAC Advisory Board was identified and a chair and co-chair for each advocacy group was selected.

The workshop closed with the program assessment and feedback conducted by Professor Richard Segal (USA). Participants provided their evaluations on the overall program, the workshop sessions, and the speakers. In addition, feedback was obtained on suggested improvements for the 2013 workshop. Before closing the workshop, delegates were invited to join one or more of the advocacy groups.

## Competing Interests

The authors have no competing interests to be declared for this manuscript.

## Declarations

The authors declare that funding for publication of the article was funded by an award provided by the National Cancer Institute (Award Number R13CA162899).

This article has been published as part of *Infectious Agents and Cancer* Volume 8 Supplement 1, 2013: Selected articles from The International Workshop on Cancer Advocacy for African Countries (CAAC) 2011. The full contents of the supplement are available online at http://www.infectagentscancer.com/supplements/8/S1 The Supplement Editors declare they have no competing interests.
